# Mesenteric embryonal rhabdomyosarcoma in an adolescent: a case report

**DOI:** 10.1093/jscr/rjad451

**Published:** 2023-08-08

**Authors:** Jackson Kakooza, Felix Odur, Esau Ogei, Katrina Taylor, Sam Kalungi, Catherine R Lewis

**Affiliations:** Department of Surgery, St. Joseph’s Hospital Kitovu, Masaka, Uganda; Department of Surgery, St. Joseph’s Hospital Kitovu, Masaka, Uganda; Department of Surgery, St. Joseph’s Hospital Kitovu, Masaka, Uganda; Department of Pathology, East Tennessee State University, Johnson City, TN, USA; Department of Pathology, Lancet Laboratories, Kampala, Uganda; Department of Surgery, St. Joseph’s Hospital Kitovu, Masaka, Uganda; Department of Surgery, East Tennessee State University, Johnson City, TN, USA

**Keywords:** rhabdomyosarcoma, mesentery, sarcoma, embryonal, adolescents

## Abstract

Rhabdomyosarcoma (RMS) is a soft tissue sarcoma that histologically resembles embryonic skeletal muscle. It can occur anywhere in the body, including tissues devoid of skeletal muscles. RMS is a common malignancy in children, and it accounts for ˃50% of all soft tissue sarcomas in children. Embryonal rhabdomyosarcoma (ERMS) mostly affects children younger than 10 years of age. The head and neck area, the genitourinary tract and the retroperitoneum are described as the preferred anatomic sites for ERMS development. However, the mesentery location is extremely rare. We report a rare case of an ERMS occurring in the mesentery of a 17-year-old male.

## INTRODUCTION

Soft tissue sarcomas have an overall incidence of 7.15% in children [1]. Within this group of neoplasms, rhabdomyosarcomas (RMS) are most common [2]. Embryonal rhabdomyosarcoma (ERMS), followed by alveolar rhabdomyosarcoma (ARMS), are common histological subtypes (~91%) [3]. RMS accounts for ˃50% of all soft tissue sarcomas in children [4]. ERMS mostly affects children younger than 10 years of age, but also occurs in adolescents and young adults [5].

RMS originates from embryonal mesenchyme that differentiates into skeletal muscle [2]. It can occur anywhere in the body, including tissues devoid of skeletal muscles. The cell of origin remains unknown. Recent evidence suggests that RMS may originate from aberrant development of non-myogenic cells [6]. The head and neck region, genitourinary tract and retroperitoneum are the most common anatomic sites for ERMS [3]. The mesenteric location is extremely rare and only a few cases are reported [7–9]. In this case report, we discuss the findings of a mesenteric RMS in an adolescent.

## CASE PRESENTATION

A 17-year-old male presented with progressive abdominal swelling for 1 month. The swelling worsened 3 days prior to admission and was associated with abdominal pain that radiated to the groins. The patient reported anorexia, nausea and vomiting. He also reported hiccups, fever, generalized body weakness and weight loss. On examination, he was afebrile with mild pallor and left inguinal lymphadenopathy. The abdomen was distended with guarding and tenderness in the right lower quadrant with an ill-defined mass measuring 6 cm × 8 cm. There were reduced bowel sounds and positive shifting dullness. Laboratory values were significant for serum sodium of 120.7 mmol/L (ref. 136–145 mmol/L) and chloride of 86.7 mmol/L (ref. 98–107 mmol/L). Complete blood count and all other serum electrolytes were within normal limits. An erect chest X-ray showed air under the diaphragm.

**Figure 1:**
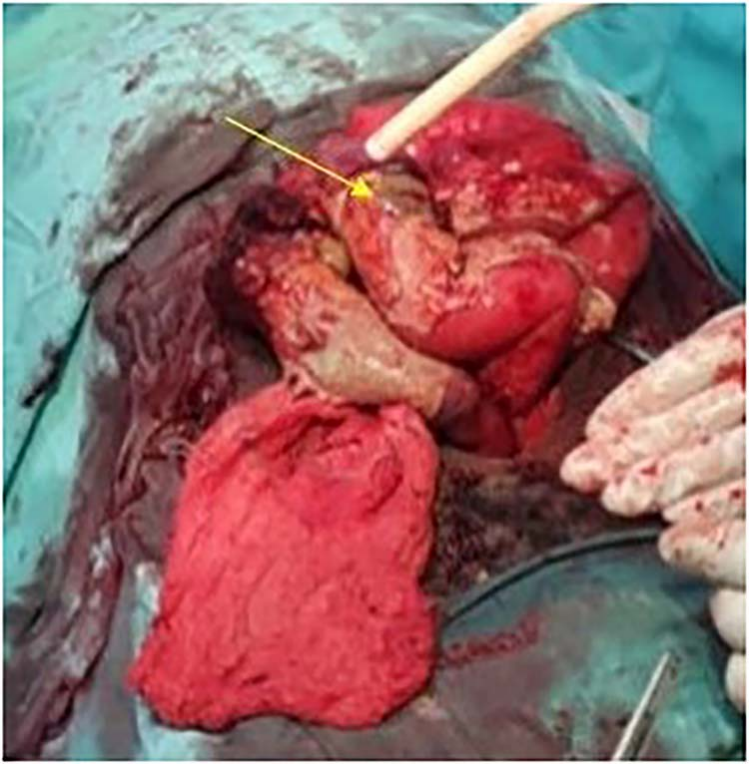
Intraoperative view showing the perforation in the ileum due to the mesenteric tumor (arrow).

The patient was taken to the operative theater. Peritoneal spillage with an ileal perforation ~40 cm from the ileocecal junction was noted (Fig. 1). A fixed, friable, tumor with neovascularization was noted involving the mesentery of the ileum. Tissue was obtained for histology. Due to the inability to completely resect the mass, the perforation was repaired primarily and an ileostomy ~30 cm from the ligament of Treitz was performed.

**Figure 2:**
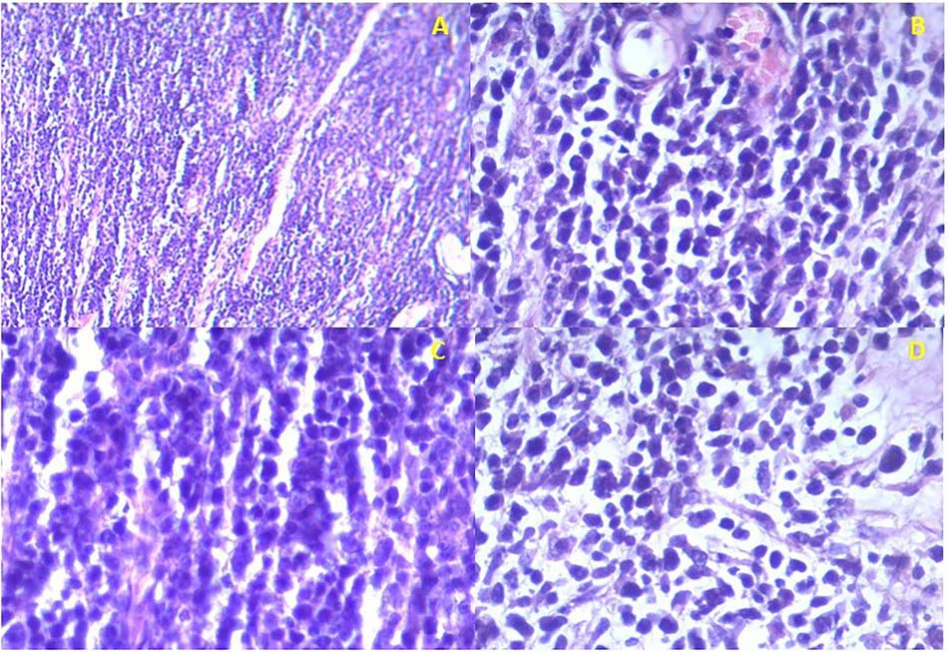
Istopathological analysis. **(a)** There is densely cellular tumor infiltrates without any discernable pattern. The intercellular matrix consists of sparse fibrous tissue. **(b)** The majority of the tumor cells appear undifferentiated with hyperchromatic oval to round nuclei with scant cytoplasm. Most of the cells are epithelioid with occasional spindle cells. **(c)** Occasional mitotic figures are seen. **(d)** Some cells have eccentric nuclei and abundant pink cytoplasm typical of rhabdomyoblasts.

Histology confirmed the presence of a high-grade malignancy. The tissue consisted of fibrous connective tissue and fat. The malignant infiltrate was composed of plump epithelioid cells and occasional spindle cells with pleomorphism. Many of the cells had eosinophilic cytoplasmic inclusions that appeared rhabdoid. Nucleoli, mitotic figures and necrosis were noted (Fig. 2). The histological features favored sarcoma with rhabdoid morphology with a provisional diagnosis of embryonal RMS. The patient was referred to the national cancer institute for further management where he expired before any intervention.

## DISCUSSION

RMS is the most common soft tissue sarcoma in children and adolescents [10]. There is slight male predominance [11, 12]. Black patients in the United States were shown to have the highest incidence of RMS [12]. In Eastern Africa, cases of RMS range from 2.6 to 16.3 per million [11]. The two most common histologic variants in children and adolescents are ERMS and ARMS. ERMS occurs in childhood with a peak age of onset ˂5 years of age [13].

Histologically, RMS can be classified into three subtypes: embryonal (67%), alveolar (32%) and pleomorphic (1%). In contrast to pleomorphic and alveolar subtypes, ERMS is more likely to present without distant metastasis [7]. Symptoms are non-specific and often depend on the location and size of the tumor [7, 10]. ERMS primarily involves the head and neck region, genitourinary tract and retroperitoneum [3]. Our patient had ERMS located at the mesentery, a rare location. Despite the diagnosis of ERMS, our patient also likely had metastasis to the inguinal lymph nodes.

RMS is difficult to diagnose due to its rarity and clinical and biological variation [14]. Imaging studies should include computed tomography scan or magnetic resonance imaging to determine the size and involvement of surrounding organs. For tumors arising from the head, imaging studies should also include full views of the neck to evaluate the cervical lymph node chains. Open biopsy of the mass is often used to confirm the diagnosis [15].

Standard chemotherapy regimens include vincristine, actinomycin-D and cyclophosphamide [16]. Agents such as carboplatin, irinotecan, topotecan and vinorelbine have shown significant efficacy in the treatment of pediatric patients with metastatic, relapsing or refractory RMS [17, 18]. Surgery is the mainstay of treatment regardless of stage or risk factors. Complete resection is indicated if possible, as it has been shown to improve survival. Margins of 0.5 cm are recommended in children as larger margins are unfeasible due to tissue limitations [16]. In our case, the patient presented late, and the tumor was unresectable.

Radiotherapy is beneficial for advanced disease. It is used to control residual bulky or microscopic tumors, especially when the tumor is unresectable [18, 19]. Surgery to resect residual disease can eliminate the need for adjuvant radiation, which is recommended for all patients except those with margin-negative RMS [14].

Historically, patients with RMS were believed to have a dismal prognosis. However, for pediatric patients with early localized disease, located in favorable sites with favorable histological types, aggressive multimodality therapy gives long-term survival rates of 90% . The prognostic risk factors include the site of the primary tumor, the extent of the initial surgical resection, age at diagnosis, histologic type, tumor-node-metastasis stage and most importantly, response to treatment. The 10-year metastasis-free survival was 72% for patients with RMS who responded to chemotherapy compared to 19% for patients who failed to respond [20]. The tumor in our case was mesenteric, a rare site, with poor prognosis.

## CONCLUSION

Rhabdomyosarcoma is prevalent in children and teenagers. There are only a few cases of embryonal rhabdomyosarcoma within the mesentery. Although uncommon, the mesentery as the primary location of this tumor should be kept in mind as a differential diagnosis for intraperitoneal tumors. Early diagnosis can lead to an increased chance of complete resection and improved prognosis.

## Data Availability

Data sharing is not applicable is not applicable to this article as no new data were created or analyzed in this study.
